# Evaluation of poor prognostic factors of respiratory related death in microscopic polyangiitis complicated by interstitial lung disease

**DOI:** 10.1038/s41598-021-81311-7

**Published:** 2021-01-15

**Authors:** Shogo Matsuda, Takuya Kotani, Takayasu Suzuka, Takao Kiboshi, Keisuke Fukui, Minako Wakama, Takaaki Ishida, Youhei Fujiki, Hideyuki Shiba, Koji Nagai, Kenichiro Hata, Takeshi Shoda, Yuri Ito, Shigeki Makino, Tohru Takeuchi

**Affiliations:** 1 Department of Internal Medicine (IV), Osaka Medical College, Daigaku-Machi 2-7, Takatsuki, Osaka 569-8686 Japan; 2 Department of Medical Statistics, Research and Development Center, Osaka Medical College, Takatsuki, Osaka Japan; 3 Department of Rheumatology, Yodogawa Christian Hospital, Osaka, Japan

**Keywords:** Immunology, Diseases, Medical research, Rheumatology, Risk factors

## Abstract

The prognosis of microscopic polyangiitis (MPA) with interstitial lung disease (ILD) is significantly worse than that of MPA without ILD. However, the clinical characteristics in MPA-ILD, especially poor prognostic factors, are not elucidated. We evaluated demographic, clinical, laboratory, and radiological findings, treatments, and outcomes of 80 patients with MPA, and investigated prognostic factors of respiratory-related death in patients with myeloperoxidase (MPO)-anti-neutrophil cytoplasmic antibody (ANCA) positive MPA-ILD. Ground-glass opacity and fibrosis were evaluated as scores on high-resolution computed tomography (HRCT). The presence of ILD was consistent with a high risk of respiratory-related death (hazard ratio, 4.8; *P* = 0.04). Multivariable logistic regression analyses using propensity scoring showed right or left lower lobe fibrosis score to be significantly associated with respiratory-related death (*P* = 0.0005 and 0.0045, respectively). A right or left lower lobe fibrosis score ≥ 2, indicating the presence of honeycombing at 1 cm above the diaphragm, was determined to be the best cut-off value indicating a poor prognosis. The 5-year survival rate was significantly lower in patients with right or left lower lobe fibrosis score ≥ 2 (survival rates: 37% and 19%, respectively) than those with a score < 2 (71% and 68%, respectively) (*P* = 0.002 and 0.0007, respectively). These findings suggest that the presence of honeycomb lesions in bilateral lower lobes on chest HRCT was associated with respiratory-related death in patients with MPO-ANCA positive MPA-ILD.

## Introduction

Microscopic polyangiitis (MPA) is an anti-neutrophil cytoplasmic antibody (ANCA) associated vasculitis (AAV) that predominantly affects small vessels with few or no immune deposits involving several organs such as skin, lung, heart, and kidney^1^. Pulmonary involvement is a common manifestation that occurs in 25% of MPA patients, and the main type of pulmonary manifestation differs between European and Asia countries^2–4^. In European countries, diffuse alveolar hemorrhage (DAH) is the most frequent pulmonary manifestation in MPA patients^4^, whereas that in Asia is ILD^2^. ILD develops more frequently in patients with myeloperoxidase (MPO)-ANCA-positive AAV than in those with proteinase3 (PR3)-ANCA-positive AAV^2^. Therefore, MPO-ANCA may be related to the pathogenesis of pulmonary fibrosis.

The prognosis of AAV patients with ILD (AAV-ILD) is significantly worse than that of AAV patients without ILD. In a Japanese nationwide AAV study, the 5-year survival rate in the patients with AAV-ILD was 50.2% versus 73.3% in those without apparent pulmonary involvement^2^. The main causes of death in MPA-ILD are respiratory infection, exacerbation of ILD, and severe vasculitis resistant to immunosuppressants^5–9^. They differ between Asian and European countries: respiratory infections and severe vasculitis such as DAH are the main causes in Asia^5,6^, whereas that in Europe is exacerbation of ILD^7–9^. However, the clinical characteristics of MPO-ANCA positive MPA-ILD, and especially factors of poor prognosis, have not been elucidated.

In this study, we retrospectively evaluated whether ILD is associated with respiratory-related death and investigated the factors of poor prognosis in MPO-ANCA positive MPA-ILD.

## Methods

### Patients

We investigated 80 patients admitted to Osaka Medical College Hospital from April 2010 to April 2019 who were diagnosed as having MPA using the Chapel Hill Consensus definition, and had positive ANCAs and/or biopsy-proven small-vessel necrotizing vasculitis^1^. Patients with malignancy, infection, drug-induced vasculitis, secondary vasculitis, vasculitis mimics, and sarcoidosis were excluded^10^. We recruited these patients from our database. All patients were hospitalized at the time of the initial remission induction therapy, and they received immunosuppressive treatments based on physician discretion. All clinical and laboratory findings, treatments and outcomes were extracted retrospectively from medical records.

This study was conducted in accordance with the Declaration of Helsinki and its amendments and was approved by Osaka Medical College and the Faculties of Medicine Ethics Committee (approval no. 1529). Informed consent was obtained from each patient.

### Clinical findings, laboratory parameters on admission

Patient demographic characteristics (age, sex, and smoking history), the period from appearance of respiratory symptoms to start of treatments, and the contents of treatments were evaluated. White blood cell (WBC) counts, hemoglobin (Hb), albumin, lactate dehydrogenase, creatinine, C-reactive protein (CRP), Krebs von den Lungen-6 (KL-6), MPO-ANCA, and PR3-ANCA were measured. Serum KL-6 level was measured using electrochemiluminescence immune assay, and serum MPO-ANCA and PR3-ANCA titers were measured by an enzyme-linked immunosorbent assay. These assays were commercially conducted by SRL (SRL Inc., Tokyo, Japan).

### Arterial blood gas analysis and pulmonary function testing (PFT)

Arterial blood gas analysis including PaO_2_, PaCO_2_, and PaO_2_/FiO_2_ (P/F) ratio was carried out in the MPO-ANCA positive MPA-ILD patients on admission. PFT parameters, including forced vital capacity (FVC), were measured by spirometry (SYSTEM21; Minato Medical Science, Osaka, Japan). Diffusion capacity of the lung for carbon monoxide was determined by the single-breath method^11–13^. All PFT results are expressed as percentages of the predicted value.

### Evaluation of high-resolution computed tomography (HRCT) scoring

**Figure 1:**
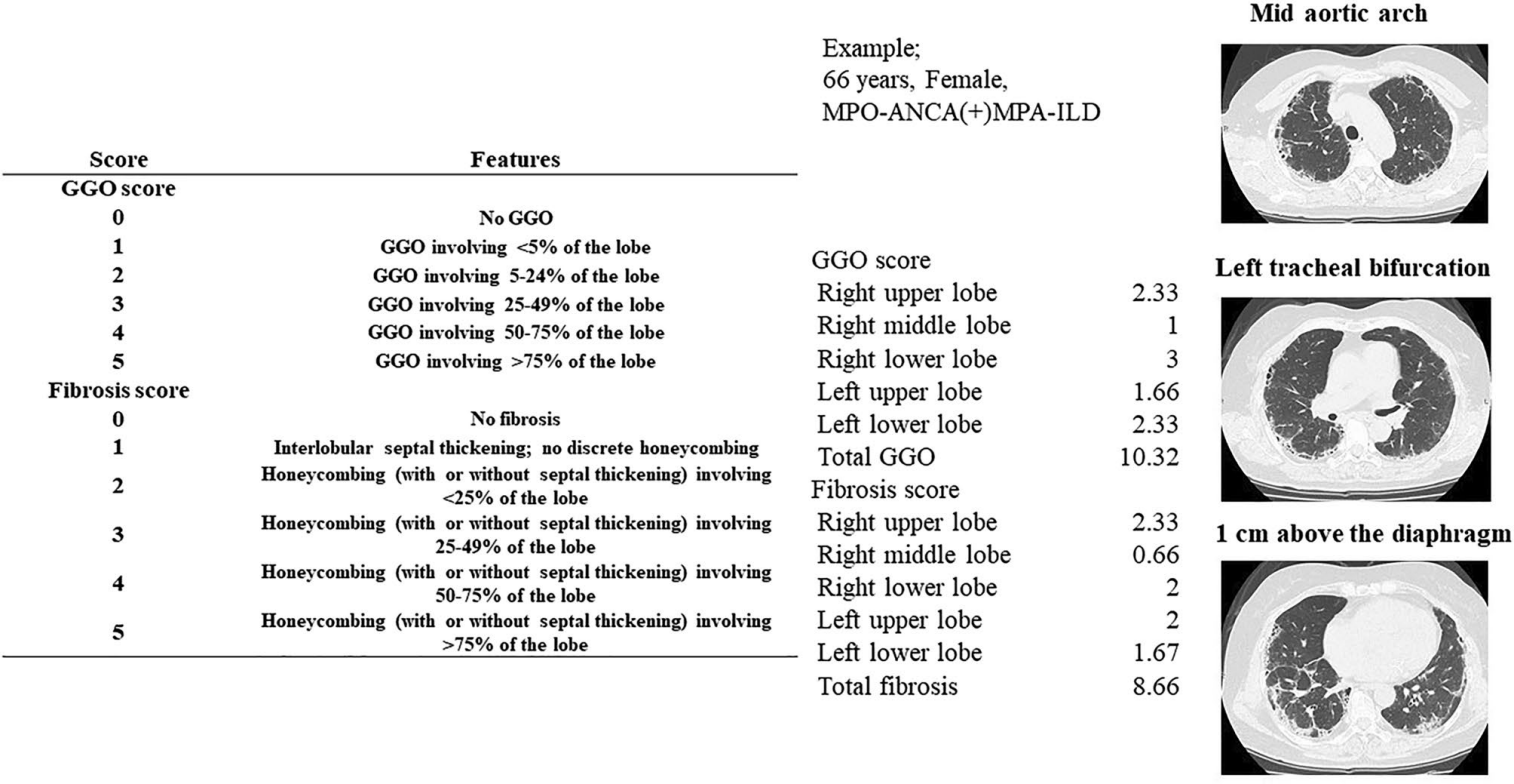
Chest HRCT scoring system using limited 3 CT levels; based on Kazerooni et al.^14^. Example: an example case of a 66-year old female with myeloperoxidase (MPO)-anti-neutrophil cytoplasmic antibody (ANCA)-positive microscopic polyangiitis (MPA) complicated by interstitial lung disease (ILD). *GGO* ground-glass opacity.

The presence of ILD was assessed by chest HRCT scans that were read by pulmonary radiologists. Prior to treatments, all patients underwent chest HRCT with a 64-detector row CT Aquilon multiscanner (Toshiba Medical System Corporation, Tokyo, Japan). HRCT was obtained with 1.0- or 1.5-mm-thick sections at 10-mm intervals throughout the entire lung. Ground-glass opacity (GGO) and fibrosis were independently evaluated as scores on HRCT images by three observers (TS, TK, and TS) blinded to the patients’ clinical information, as previously described^14^. Limited CT images, taken at the levels of the mid-aortic arch, left tracheal bifurcation, and 1 cm above the diaphragm, were scored. Each lobe (right upper, middle, and lower lobes, and left upper and lower lobes) of the lungs was semiquantitatively scored at the three sites on a scale from 0 to 5 for GGO and for septal thickening and honeycombing, as follows: scores for GGO involving the lobe (0, none; 1, < 5%; 2, 5% to < 25%; 3, 25–49%; 4, 50–75%; and 5, > 75%) and fibrosis scores for honeycombing involving the lobe (0, none; 1, interlobular septal thickening without discrete honeycombing; 2, < 25%; 3, 25–49%; 4, 50–75%; and 5, > 75%)^15^. The average score was summed as the total HRCT score. The evaluation method and the concrete example of chest HRCT scoring are shown in Fig. 1.

### Evaluation of disease severity and outcome

Disease severity was determined according to the European Vasculitis Study Group (EUVAS) categorization system^16^. Organ involvement was evaluated according to the Birmingham Vasculitis Activity Score (BVAS), version 3^17^. The 2009 five-factor score (FFS), which is used to evaluate prognosis at the diagnosis of MPA, was evaluated for each patient^18^. Respiratory-related mortality rate and underlying cause of death, defined using the International Statistical Classification of Diseases and Related Health Problems 10th Revision (ICD-10) were obtained from our database.

### Statistical analysis

Data are presented as the median (range). Fisher’s exact test was used when appropriate, and the Wilcoxon signed-rank test was used for the comparison of median values. *P* values of < 0.05 were considered statistically significant. The Kaplan–Meier method was used to assess survival curves and the log-rank test to evaluate the significance of differences between two groups. Survival time was calculated from the date of remission induction therapy and ended at the latest hospital visit, the date of censoring, or the time of respiratory-related death. Cox proportional hazards models were also used to evaluate relative risk of death between two groups.

We used multivariate Cox regression analysis to compare the prognosis between MPA without ILD and MPA-ILD. We selected as covariates the percentage of kidney involvement and BVAS.

Risk factors associated with respiratory-related death yielding significance values of *P* < 0.05 in univariate analysis were extracted and were assessed using univariate and multivariate logistic regression analysis.

We selected as covariates age, sex, history of smoking, initial oral prednisolone dose, and duration of ILD. Because of the low number of respiratory-related deaths, propensity score adjustment was used to reduce covariates to a single variable. The propensity score was computed separately for each candidate risk factor and then used as a covariate in the model evaluating the adjusted hazard ratio (HR)^19^.

We used propensity score adjustment for each of the above risk factors in the multivariate model. All risk factors were continuous variables, and they were divided into two groups by the median of risk factors for calculating propensity scores with a binary logistic regression. For example, when the adjusted effect of %FVC was evaluated, a propensity score was created to provide the predicted probability of %FVC as a function of the above covariates.

We compared the demographic and background characteristics between two groups in a univariate analysis, and then we estimated the HRs of patient outcomes in a univariate and multivariate analysis with a Cox regression model. Receiver operating characteristic (ROC) curve analysis was used to determine the most suitable cut-off level for predicting the prognosis of MPO-ANCA positive MPA-ILD. The Skillings-Mack test was used to investigate the statistical difference in the HRCT scores between the initial measurement and those at 2 and 12 months after treatment initiation. Dunn’s post hoc test was used for multiple comparisons. The data were analysed using JMP version 14.0 (SAS Institute Inc., Cary, NC, USA). R 3.6.0 (The R Foundation for Statistical Computing, Vienna, Austria) was used for the Skillings-Mack test.

## Results

### Patient profiles

Eighty MPA patients were enrolled in our study. MPO-ANCA was positive in 79 patients, whereas PR3-ANCA was positive in 3 patients. Two patients were double positive for MPO/PR3-ANCA. Of the 80 patients with MPA, 47 had ILD. Sixteen patients were diagnosed as having ILD at the time of diagnosis, and 31 patients developed ILD a median 24 months before the diagnosis of MPA. After a median follow-up period of 32.9 months, 24 patients died. 14 patients died due to respiratory-related death, and 10 patients die due to non-respiratory death. The causes of non-respiratory-related deaths included cardiac failure (n = 2), cerebral hemorrhage (n = 1), cerebral infarction (n = 1), mediastinal hematoma (n = 1), gastrointestinal hemorrhage (n = 1), and others (n = 4).

We compared the baseline clinical characteristics between survival and non-survival patients in Table 1. The mean age, serum levels of creatinine, and serum CRP levels tended to be higher in non-survivors than in survivors; however, there were no significant differences between the two groups (*P* = 0.05, 0.10, and 0.17, respectively). The percentage of FFS ≦ 1 was significantly lower in the non-survivor group than in the survivor group (*P* = 0.008), and the percentage of “severe,” as defined by the EUVAS classification, was significantly higher in the non-survivor group than in the survivor group (*P* = 0.04).

**Table 1:** Comparison of clinical characteristics, and outcomes between non-survivors and survivors in MPA patients.

Characteristics	Non-survivors (n = 24)	Survivors (n = 56)	*P* value
Age, years	76 (70–81)	73 (68–78)	0.054
Female, n (%)	13 (54)	29 (52)	1.000
**Laboratory findings**			
WBC, /mm^3^	12,930 (8010–16,115)	10,715 (7265–13,988)	0.24
Hb, g/dL	10.5 (8.2–12.5)	10.1 (8.7–12)	0.64
Alb, g/dL	2.5 (2.0–3.1)	2.5 (2.2–3.2)	0.72
LD, IU/L	206 (170–244)	192 (166–237)	0.73
Cr, mg/dL	1.6 (0.8–2.3)	1.1 (0.7–1.8)	0.10
CRP, mg/mL	11.4 (4.3–14.3)	9.3 (3.0–12.4)	0.17
Positive anti-MPO-ANCA, n (%)	23 (95.8)	56 (100)	0.3
Positive anti-PR3-ANCA, n (%)	2 (8.3)	1 (1.8)	0.21
MPO-ANCA titer, U/mL	80.3 (35.4–245)^a^	97.9 (58.5–253.3)	0.70
BVAS at onset	20 (13–23)	16.5 (9.3–22.8)	0.26
**Five factor score 2009**			
≦ 1	1 (4.2)	18 (32.1)	0.008**
2	16 (66.7)	29 (51.8)	0.325
≧ 3	7 (29.2)	9 (16.1)	0.226
**EUVAS-defined disease severity**			
Localized	0 (0)	3 (5.4)	0.55
Early systemic	2 (8.3)	10 (17.9)	0.33
Systemic	13 (54.2)	34 (60.7)	0.63
Severe	9 (37.5)	9 (16.1)	0.04*
**Outcome**			
Respiratory related death, n (%)	14 (58.3)		
**Cause of non-respiratory death**			
Cardiac failure, n (%)	2 (8.3)		
Cerebral hemorrhage, n (%)	1 (4.2)		
Cerebral infarction, n (%)	1 (4.2)		
Mediastinal hematoma, n (%)	1 (4.2)		
Gastrointestinal hemorrhage, n (%)	1 (4.2)		
Others, n (%)	4 (16.7)		

The laboratory markers are presented as the median (interquartile range). The P-values were estimated using Fisher’s exact test or Wilcoxon rank sum test.

*MPA* microscopic polyangiitis, *WBC* white blood cell, *Hb* hemoglobin, *Alb* albumin, *LD* lactate dehydrogenase, *Cr* creatinine, *CRP* C-reactive protein, *MPO-ANCA* myeloperoxidase-anti-neutrophil cytoplasmic autoantibody, *PR3-ANCA* proteinase 3-anti-neutrophil cytoplasmic antibody, *BVAS* Birmingham Vasculitis Activity Score, *EUVAS* European Vasculitis Study Group.

*P < 0.05, **P < 0.01.

^a^Number of subjects, n = 23.

### Comparison of clinical characteristics and treatment between MPA with or without ILD

Table 2 shows the clinical characteristics and treatment contents between the 47 MPA patients with ILD and the 33 MPA patients without ILD. There were no significant differences between the MPA patients with and without ILD in proportion of females, and frequency of smoking history. The median age was higher in the MPA-ILD group (76 [70–80] years) than in the MPA without ILD group (71 [67–78] years) (*P* = 0.036). The serum levels of creatinine were significantly higher in the MPA without ILD group (1.7 [1.1–4.6] mg/dL) than in the MPA-ILD group (0.9 [0.7–1.5] mg/dL) (*P* = 0.0016). The initial serum levels of KL-6 were significantly higher in the MPA-ILD group (383 [245–668] U/mL) than those in the MPA without ILD group (174 [120–242] U/mL) (*P* < 0.0001). The total BVAS was significantly higher in MPA without ILD group (21 [13.5–25]) than that in the MPA-ILD group (14 [8–21]) (*P* = 0.006). There were no significant differences in the FFS 2009 and the frequency of EUVAS-defined disease severity between the two groups. Although the total dose of intravenous cyclophosphamide (IVCY) was higher in the MPA-ILD group (*P* = 0.013), there were no significant differences in either the doses of prednisolone or frequency of administration of IVCY. More details on the frequency of systemic symptoms and treatments are provided online in Supplementary Table S1.

**Table 2:** Comparison of clinical characteristics, and outcomes of patients between MPA with or without ILD.

Characteristics	MPA without ILD (n = 33)	MPA with ILD (n = 47)	*P* value
Age, years	71 (67–78)	76 (70–80)	0.036*
Female, n (%)	18 (55)	24 (51)	0.822
Smoking history, n (%)	17 (51.5)	27 (57.5)	0.652
**Laboratory findings**			
WBC, /mm^3^	12,500 (8135–14,625)	10,860 (7310–14,190)	0.458
Hb, g/dL	9.4 (8.0–11.4)	10.3 (8.6–12.3)	0.132
Alb, g/dL	2.3 (2–3.1)	2.7 (2.2–3.3)	0.08
LD, IU/L	197 (168–228)	194 (165–253)	0.458
Cr, mg/dL	1.7 (1.1–4.6)	0.9 (0.7–1.5)	0.0016**
CRP, mg/mL	11.6 (3.1–14.7)	8.4 (3.0–12.2)	0.14
Positive anti-MPO-ANCA, n (%)	32 (97)	47 (100)	0.413
Positive anti-PR3-ANCA, n (%)	1 (3.0)	2 (4.3)	1.00
MPO-ANCA titer, U/mL	144 (52–289)	94.1 (49.5–235.1)	0.15
KL-6, U/mL	174 (120–242)	383 (245–668)	< 0.0001***
BVAS at onset	21 (13.5–25)	14 (8–21)	0.006**
**Five factor score 2009**			
≦ 1	8 (24.2)	11 (23.4)	1.000
2	16 (48.5)	29 (61.7)	0.262
≧ 3	9 (27.3)	7 (14.9)	0.256
**EUVAS-defined disease severity**			
Localized	1 (3.03)	2 (4.26)	1.000
Early systemic	4 (12.1)	8 (17.0)	0.75
Systemic	20 (60.6)	27 (57.5)	0.821
Severe	8 (24.2)	10 (21.3)	0.791
**Outcome**			
Respiratory related death, n (%)	2 (6.1)	12 (25.5)	0.035*
Others, n (%)	8 (24.2)	2 (4.3)	
**Cause of respiratory-related death**			
Infectious pneumonia	1 (3.0)	9 (19.2)	0.041*
Diffuse alveolar hemorrhage	1 (3.0)	2 (4.3)	1.000
Exacerbation of ILD	0 (0)	1 (2.1)	1.000

The laboratory markers are presented as the median (interquartile range). The P-values were estimated using Fisher’s exact test or Wilcoxon rank sum test.

*MPA* microscopic polyangiitis, *ILD* interstitial lung disease, *WBC* white blood cell, *Hb* hemoglobin, *Alb* albumin, *LD* lactate dehydrogenase, *Cr* creatinine, *CRP* C-reactive protein, *MPO-ANCA* myeloperoxidase-anti-neutrophil cytoplasmic autoantibody, *PR3-ANCA* proteinase 3-anti-neutrophil cytoplasmic antibody, *KL-6* Krebs von den Lungen-6, *BVAS* Birmingham Vasculitis Activity Score, *EUVAS* European Vasculitis Study Group.

*P < 0.05, **P < 0.01, ***P < 0.001.

### Incidence of respiratory-related deaths

During follow-up period, 14 out of 80 patients died due to respiratory-related death. Twelve of the 14 patients had ILD. The incidence rate of respiratory-related death was 5.3 per 100 patient-years. The mean age of those having respiratory-related death was 77.5 years, and 9 patients (64.3%) were men. The causes of respiratory-related death were infectious pneumonia in 10 patients, including bacterial pneumonia (8 patients), tuberculous pneumonia (1 patient), and cytomegalovirus pneumonia (1 patient), DAH in 3, and exacerbation of ILD in 1 (Table 2).

### Comparison of prognosis in MPA patients with and without ILD

**Figure 2:**
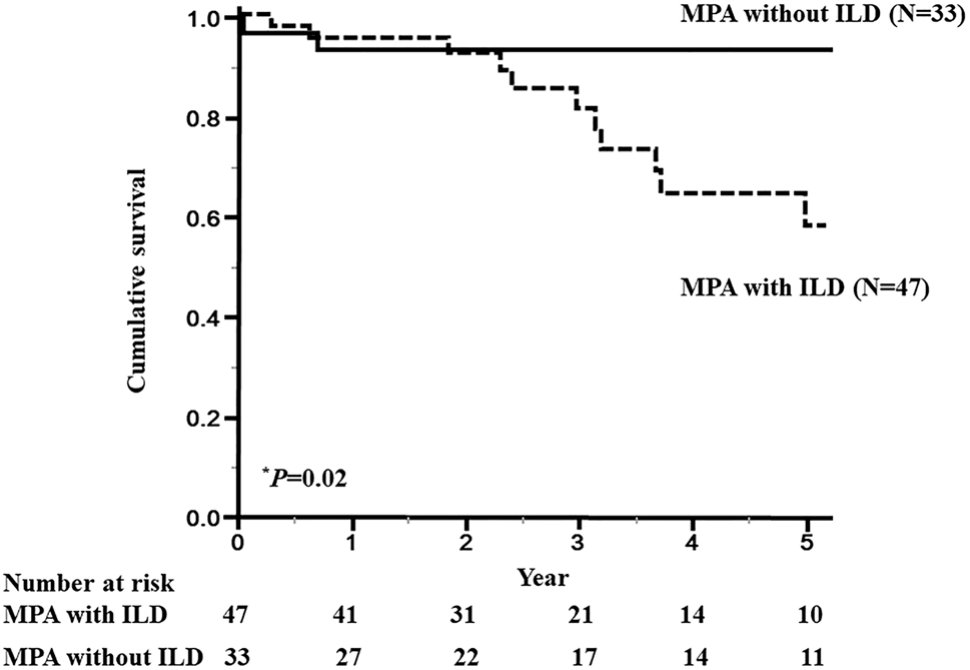
Survival curves of patients with microscopic polyangiitis (MPA) based on the presence of interstitial lung disease (ILD). The 5-year survival rate for MPA patients with ILD (58%) was significantly lower than that for MPA patients without ILD (93%) (*P* = 0.02). Solid line: MPA patients without ILD, dashed line: MPA patients with ILD. Survival rates were calculated by the Kaplan–Meier method and compared by log-rank test. *P < 0.05.

To investigate the prognosis of MPA patients with and without ILD, Kaplan–Meier survival curves were plotted based on the ILD. The 5-year survival rate was significantly lower for the patients with ILD (58%) than that for the patients without ILD (93%) (*P* = 0.02) (Fig. 2). In univariate Cox regression analysis, the presence of ILD was also consistent with a high risk of respiratory-related death (HR 4.8; 95% confidence interval 1.1–21.4; *P* = 0.04). In multivariate Cox regression analysis, the presence of ILD was independently consistent with a high risk of respiratory-related death (HR 7.2; 95% confidence interval 1.5–34.8; *P* = 0.01).

### Comparison of clinical features between survivors and non-survivors due to respiratory-related diseases in MPO-ANCA positive MPA-ILD

To identify the poor prognostic factors of MPO-ANCA positive MPA-ILD, we compared baseline clinical and laboratory findings between the 35 MPA-ILD survivors and the 12 MPA-ILD non-survivors (Table 3). There were no significant differences in age, sex, time from onset of respiratory symptoms to treatment initiation, and history of smoking or in the rate of DAH, ANCA phenotype, and initial laboratory findings. In the PFT results, %FVC was significantly lower in the non-survivors (73.4% [66.6–79.6]) than that in the survivors (91.9% [79.9–96.1]) (*P* = 0.0078). The total GGO and fibrosis scores of chest HRCT were significantly higher in the non-survivors than those in the survivors (*P* = 0.047, and 0.016, respectively). The right middle/lower lobe and the left lower lobe fibrosis scores were significantly higher in the non-survivors than those in the survivors (*P* = 0.041, 0.0019, and 0.024, respectively). More details on disease severity and treatments are provided online in Supplementary Table S2.

**Table 3:** Comparison of clinical characteristics, and contents of treatment between survivors and non-survivors in MPA with ILD.

Characteristics	Survivors (n = 35)	Non-survivors (n = 12)	*P* value
Age, years	73 (70–78)	78 (71–86)	0.236
Female, n (%)	20 (57.1)	4 (33.3)	0.193
Disease duration, months	2 (0–36)	19 (0–42)	0.485
Smoking history, n (%)	21 (60.0)	6 (50)	0.737
DAH, n (%)	5 (14.3)	2 (16.7)	1.000
**Laboratory findings**			
WBC, /mm^3^	10,470 (7000–14,900)	12,110 (7903–13,663)	0.932
Hb, g/dL	10.3 (8.6–12.2)	10.7 (8.8–12.8)	0.678
Alb, g/dL	2.9 (2.3–3.3)	2.4 (2–3.4)	0.348
LD, IU/L	206 (167–262)	186 (145–243)	0.373
Cr, mg/dL	0.9 (0.7–1.34)	1.1 (0.7–1.7)	0.49
CRP, mg/mL	7.9 (3.0–12.2)	9.7 (2.3–14.0)	0.393
MPO-ANCA titer, U/mL	95.2 (49.5–235.1)	62.3 (36.0–239.5)	0.51
KL-6, U/mL	371 (245–626)	415 (240.3–675.3)	0.696
PaO_2_/FiO_2_ ratio	406.7 (352.9–441.9)	396.2 (341.5–433.3)	0.733
**PFT findings**			
%FVC	91.9 (79.9–96.1)^a^	73.4 (66.6–79.6)^b^	0.0078**
FEV_1_/FVC	81.0 (77.0–86.2)^a^	86.3 (78.3–91.7)^b^	0.163
%Dlco, ml/min/mmHg	43.5 (34.9–51.5)^c^	31.4 (24.2–57.1)^d^	0.39
**Chest HRCT score**			
**GGO score**			
Right upper lobe	0.66 (0.33–1.33)	1.33 (0.75–2)	0.068
Right middle lobe	0.33 (0–0.67)	0.83 (0.08–1)	0.219
Right lower lobe	1.67 (1.33–2)	2 (2–2.83)	0.082
Left upper lobe	0.66 (0–1.3)	1 (1–1.92)	0.082
Left lower lobe	1.33 (0.66–2)	1.66 (1.08–2)	0.396
Total	4.98 (2.99–7.64)	6.66 (5.17–8.66)	0.047*
**Fibrosis score**			
Right upper lobe	0.66 (0.33–1.33)	1 (0.66–1.66)	0.149
Right middle lobe	0 (0–0.66)	0.66 (0.33–1)	0.041*
Right lower lobe	1.33 (1–1.66)	2 (1.66–2.25)	0.0019**
Left upper lobe	0.66 (0.33–1.33)	1 (0.66–1.66)	0.283
Left lower lobe	1.33 (0.66–1.66)	1.83 (1.33–2)	0.024*
Total	4 (2.98–6.65)	6.16 (5.32–8.81)	0.016*

The laboratory markers are presented as the median (interquartile range). The P-values were estimated using Fisher’s exact test or Wilcoxon rank sum test.

*MPA* microscopic polyangiitis, *ILD* interstitial lung disease, *DAH* diffuse alveolar hemorrhage, *WBC* white blood cell, *Hb* hemoglobin, *Alb* albumin, *LD* lactate dehydrogenase, *Cr* creatinine, *CRP* C-reactive protein, *MPO-ANCA* myeloperoxidase-anti-neutrophil cytoplasmic autoantibody, *KL-6* Krebs von den Lungen-6, *PaO*_*2*_*/FiO*_*2*_* ratio* arterial partial pressure of O_2_ and the fraction of inspired oxygen ratio, *PFT* pulmonary function test, *FVC* forced vital capacity, *FEV* forced expiratory volume in one second, *Dlco* diffusing capacity of the lung for carbon monoxide, *HRCT* high resolution computed tomography, *GGO* ground-glass opacity.

*P < 0.05, **P < 0.01.

^a^Number of subjects, n = 26.

^b^Number of subjects, n = 11.

^c^Number of subjects, n = 25.

^d^Number of subjects, n = 9.

### Cox regression analysis of respiratory-related deaths in MPO-ANCA positive MPA-ILD

Percent FVC, total GGO score, right middle lobe fibrosis score, right lower lobe fibrosis score, left lower lobe fibrosis score, and total fibrosis score were extracted as risk factors in this study. To confirm these findings, we next performed univariate and multivariate analyses using Cox regression analysis. A univariate analysis with a Cox regression model showed that %FVC, total GGO score, right or left lower lobe fibrosis score, and total fibrosis score were predictors of respiratory-related death in MPA with ILD (*P* = 0.02, 0.02, < 0.0001, 0.01, and 0.02, respectively).

**Figure 3:**
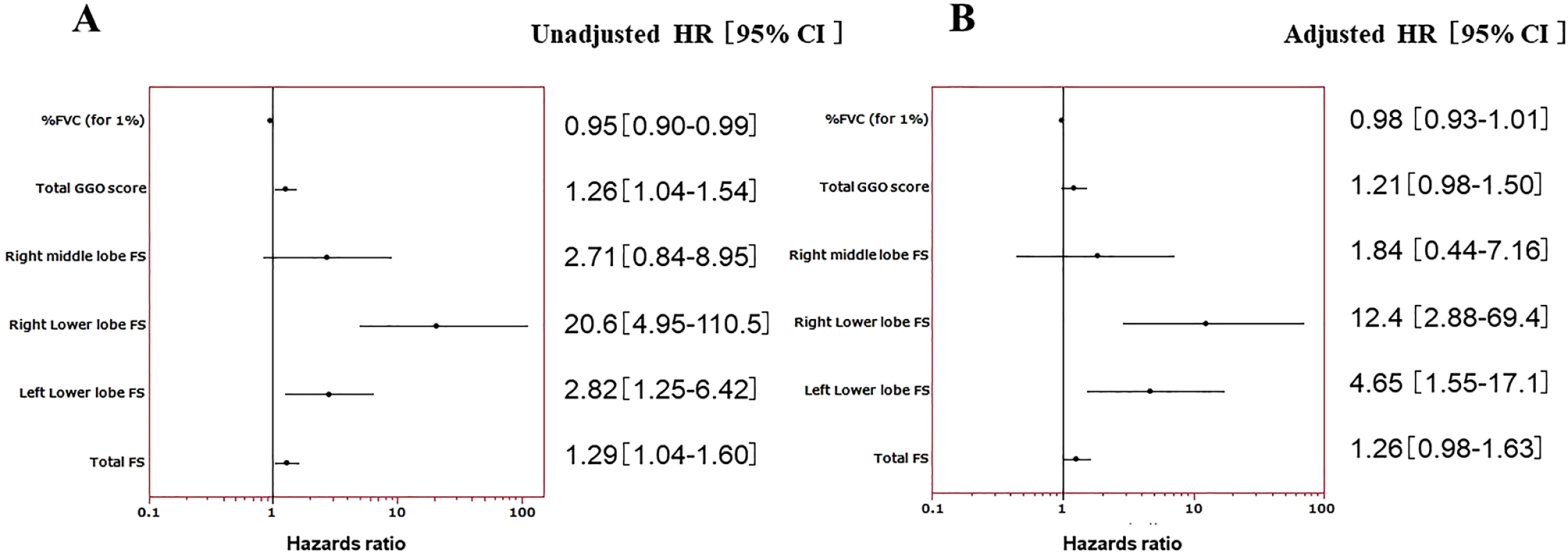
Forest plots analyzing the predictive values of risk factors for respiratory-related death. Solid squares with bars represent the hazard ratios with 95% confidence intervals. (**A**) Univariate analysis with a Cox regression model. (**B**) Multivariate analysis with a Cox regression model using propensity scoring to control for age, sex, smoking history, initial prednisolone dose, and disease duration.

After adjusting for covariates and using propensity scoring to control for age, sex, smoking history, initial prednisolone dose, and disease duration, multivariate analysis with a Cox regression model revealed right or left lower lobe fibrosis score to be risk a factor for poor prognosis (*P* = 0.0005, and 0.0045, respectively) (Fig. 3). Detailed HR and 95% confidence interval data are provided in Supplementary Table S3.

### Cut-off levels of HRCT score and survival rates

To estimate the cut-off points for assessing factors related to the poor prognosis of MPO-ANCA positive MPA-ILD, ROC curve analysis was carried out using the fibrosis score of the right and left lower lobes. The level that maximized the area under the ROC curve was 2 for the right lower lobe fibrosis score (sensitivity: 67%, specificity: 78%) and 2 for the left lower lobe fibrosis score (sensitivity: 50%, specificity: 88%). Thus, a right or left lower lobe fibrosis score ≥ 2, indicating the presence of honeycombing at 1 cm above the diaphragm, was the best cut-off level for indicating a poor prognosis.

**Figure 4:**
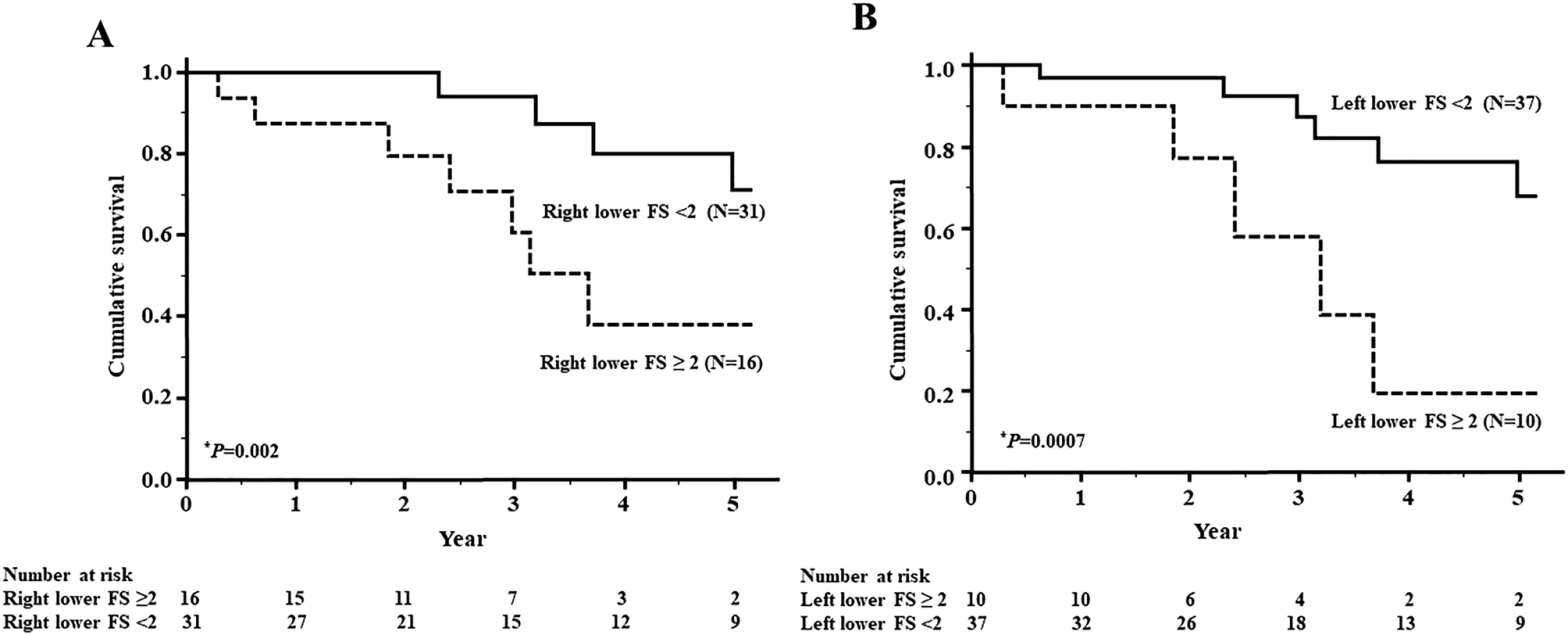
Survival curves of patients with microscopic polyangiitis (MPA)-interstitial lung disease (ILD) based on their right or left lower lobe fibrosis score. (**A**) The 5-year survival rate was significantly lower in the patients with a right lower lobe fibrosis score ≥ 2 (37%) than in those with a score < 2 (71%) (*P* = 0.002). Solid line: < 2, dashed line: ≥ 2. (**B**) The 5-year survival rate was significantly lower in the patients with a left lower lobe fibrosis score ≥ 2 (19.3%) than in those with a score < 2 (68%) (*P* = 0.0007). Solid line: < 2, dashed line: ≥ 2. Survival rates were calculated by the Kaplan–Meier method and compared by log-rank test. *P < 0.05.

The patients were then divided into two groups based on these cut-off levels, and Kaplan–Meier survival curves were plotted (Fig. 4A,B). The 5-year survival rate was significantly lower in both the patients with a right lower lobe fibrosis score ≥ 2 (37%) versus a score < 2 (71%) (*P* = 0.002) and in those with a left lower lobe fibrosis score ≥ 2 (19.3%) versus a score < 2 (68%) (*P* = 0.0007).

### Serial change of HRCT scores in MPO-ANCA positive MPA-ILD initially and at 2 and 12 months after immunosuppressive treatment

**Figure 5:**
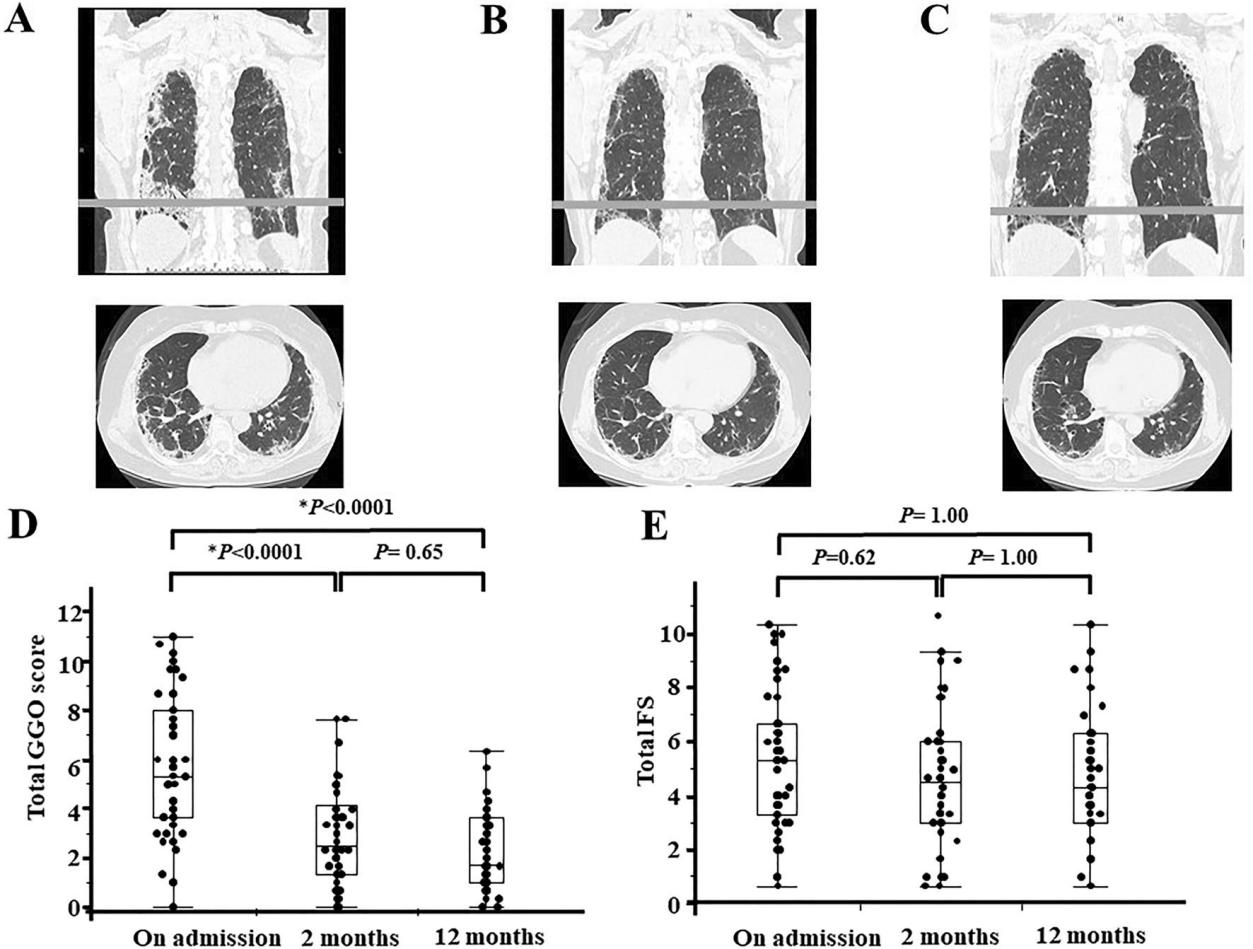
Serial change of chest high-resolution computed tomography findings (HRCT) and HRCT scores in microscopic polyangiitis (MPA)-interstitial lung disease (ILD). (**A**) Typical chest HRCT findings in MPA-ILD before immunosuppressive therapy. (**B**) Typical chest HRCT findings in MPA-ILD 2 months after immunosuppressive therapy. (**C**) Typical chest HRCT findings in MPA-ILD 12 months after immunosuppressive therapy. Upper image is a coronal section and the lower image is an axial section. The gray lines are located 1 cm above the diaphragm. (**D**) Total ground-glass opacity (GGO) score significantly decreased from the initial measurement to 2 months after starting immunosuppressive therapy (*P* < 0.0001) and sustained the same levels from 2 to 12 months. (**E**) There were no significant differences in total fibrosis score (FS) between the initial measurement and those at 2 and 12 months. Statistical analysis was carried out with the Skillings–Mack test and a Dunn’s post hoc test for multiple comparison. *P < 0.05.

Next, we examined serial changes in chest HRCT score, which are shown in Fig. 5. We followed up the patients with HRCT scans performed at 2 and 12 months after immunosuppressive treatment. We followed up 46 patients at 2 months and 39 patients at 12 months. Total GGO scores and total fibrosis scores changed with different patterns. Total GGO scores at 2 months after starting immunosuppressive therapy were significantly decreased compared with those at the initial measurement (*P* < 0.0001) and were sustained the same levels from 2 to 12 months (Fig. 5D). In contrast, there were no significant differences in the total fibrosis scores between the initial measurement and those at 2 and 12 months (Fig. 5E).

## Discussion

The present study showed that the prognosis of MPO-ANCA positive MPA-ILD was poor compared with that of MPA without ILD. The main causes of death were respiratory infection and DAH in MPO-ANCA positive MPA-ILD. Several biomarkers such as CRP, MPO-ANCA, and KL-6, BVAS, and PFT findings were not different between survivors and non-survivors of MPO-ANCA positive MPA-ILD, whereas bilateral lower lobe fibrosis scores were significantly higher in the non-survivors. ROC curve analysis showed that the cut-off level for indicating a poor prognosis was 2 for fibrosis score in the bilateral lower lobes. Thus, we showed that the fibrosis scores in the bilateral lower lobes were poor prognostic factors in MPO-ANCA positive MPA-ILD.

HRCT scoring is a useful method for evaluating the extent of ILD, including idiopathic and connective tissue disease-related ILD^14,20^. Suzuki et al. reported that total GGO and fibrosis scores were significantly higher in MPA-ILD than in MPA without ILD^5^, but the relationship between HRCT scores and outcomes in MPO-ANCA positive MPA-ILD were not elucidated. In our study, high bilateral lower lobe fibrosis scores ≥ 2 were related to respiratory-related death. Thus, HRCT scoring could be a useful tool for evaluating the prognosis of MPO-ANCA positive MPA-ILD.

A fibrosis score of ≥ 2 in this study indicates the presence of honeycombing at 1 cm above the diaphragm. Honeycombing represents an irreversible structural lung abnormality, and the extension of honeycomb lesions due to ILD progression causes respiratory failure^21^. Also, respiratory infections occurring in patients with honeycomb lesions are related to respiratory-related death^22,23^. Therefore, the survival rate of MPO-ANCA positive MPA-ILD patients with honeycomb lesions was lower than that of patients without honeycomb lesions^6^. These previous reports supported our results.

Contrastingly, GGOs represent an acute inflammation process in MPO-ANCA positive MPA-ILD, and correspond to alveolar hemorrhage, interstitial chronic inflammation in the alveolar septa, and vasculitis in small-sized arteries with infiltration of lymphocytes in the pathologic findings of MPO-ANCA-positive AAV-ILD^24,25^. GGOs on chest HRCT respond well to immunosuppressive therapy and disappear after treatment^24^. Therefore, the GGO score in chest HRCT may not be associated with respiratory-related death in MPO-ANCA positive MPA-ILD.

Previous reports showed that Hb and CRP, MPO-ANCA titer, and revised FFS were not different between MPA-ILD and MPA without ILD^2,5,7,8^. This result generally correlates with our study. In our study, BVAS was significantly lower in the MPA-ILD group than the MPA without ILD group. However, the 5-year survival rate was significantly lower in the MPA-ILD group than the MPA without ILD group perhaps because ILD was not included in the BVAS^16^. Total BVAS correlated with a poor prognosis in MPA^26^, but BVAS didn’t predict respiratory-related death in MPO-ANCA positive MPA-ILD. Also, the serum KL-6 level was significantly higher in the MPO-ANCA positive MPA-ILD patients than in those without ILD, but there were no significant differences between the survival and non-surviving groups with MPO-ANCA positive MPA-ILD. Additionally, %FVC was significantly lower in the non-surviving group than in the survival group in MPO-ANCA positive MPA-ILD, although multivariate analysis showed no significant difference. Our results showed that these biomarkers do not accurately predict a poor prognosis of MPO-ANCA positive MPA-ILD.

IVCY was administered at 0.5 g/m^2^ every monthly during induction remission therapy at our faculty, and the IVCY dose was reduced to 75–50% for MPA patients who have kidney failure according to the Japanese patients with MPO-ANCA-associated vasculitis study^27^. Serum creatinine levels were significantly higher in patients with MPA without ILD than in patients with MPA-ILD in our study; therefore, the cumulative dose of IVCY was lower in patients with MPA without ILD than in patients with MPA-ILD, although there were no significant differences in the frequency of administration of IVCY between them.

Our study has several limitations. It is a retrospective, single-center study involving a small number of patients, and thus, selection bias may be present. Our data may also be affected by indication bias because treatments for MPO-ANCA positive MPA-ILD were determined at the physician’s discretion. Moreover, ICD-10 has a limitation because the clinical terms in this classification system may not accurately capture the disease^28^.

## Conclusions

In conclusion, the prognosis of MPO-ANCA positive MPA-ILD was poor and the causes of death are mainly pulmonary infections and DAH in Japanese patients. A high fibrosis score for, and especially the presence of honeycomb lesions in, the right or left lower lobe on chest HRCT was significantly associated with respiratory-related death in patients with MPO-ANCA positive MPA-ILD. Further investigations are needed to evaluate the prognosis of MPO-ANCA positive MPA-ILD and clarify useful markers of disease progression, activity, and prognosis in MPO-ANCA positive MPA-ILD.

## Supplementary Information

Supplementary Information.

## References

[1] Jennette JC (2013). 2012 revised international chapel hill consensus conference nomenclature of vasculitides. Arthritis Rheum..

[2] Hirayama K (2015). Pulmonary involvements of anti-neutrophil cytoplasmic autoantibody-associated renal vasculitis in Japan. Nephrol. Dial. Transplant..

[3] Agard C, Mouthon L, Mahr A, Guillevin L (2003). Microscopic polyangiitis and polyarteritis nodosa: How and when do they start?. Arthritis Rheum..

[4] Guillevin L (1999). Microscopic polyangiitis: Clinical and laboratory findings in eighty-five patients. Arthritis Rheum..

[5] Suzuki A (2019). Chest high-resolution CT findings of microscopic polyangiitis: A Japanese first nationwide prospective cohort study. AJR Am. J. Roentgenol..

[6] Zhao W (2019). Clinical features and prognosis of microscopic polyangiitis with usual interstitial pneumonia compared with idiopathic pulmonary fibrosis. Clin. Respir. J..

[7] Comarmond C (2014). Pulmonary fibrosis in antineutrophil cytoplasmic antibodies (ANCA)-associated vasculitis: A series of 49 patients and review of the literature. Medicine (Baltimore).

[8] Tzelepis GE (2010). Prevalence and outcome of pulmonary fibrosis in microscopic polyangiitis. Eur. Respir. J..

[9] Hervier B (2009). Pulmonary fibrosis associated with ANCA-positive vasculitides. Retrospective study of 12 cases and review of the literature. Ann. Rheum. Dis..

[10] Watts R (2007). Development and validation of a consensus methodology for the classification of the ANCA-associated vasculitides and polyarteritis nodosa for epidemiological studies. Ann. Rheum. Dis..

[11] Standardization of spirometry—1987 update. Statement of the American Thoracic Society. *Am. Rev. Respir. Dis.* **136**, 1285–1298 (1987). 10.1164/ajrccm/136.5.1285 3674589

[12] Single breath carbon monoxide diffusing capacity (transfer factor). Recommendations for a standard technique. Statement of the American Thoracic Society. *Am. Rev. Respir. Dis.* **136**, 1299–1307 (1987). 10.1164/ajrccm/136.5.1299 3674590

[13] Lung function testing: selection of reference values and interpretative strategies. American Thoracic Society. *Am. Rev. Respir. Dis.* **144**, 1202–1218 (1991). 10.1164/ajrccm/144.5.1202 1952453

[14] Kazerooni EA (1997). Thin-section CT obtained at 10-mm increments versus limited three-level thin-section CT for idiopathic pulmonary fibrosis: Correlation with pathologic scoring. AJR Am. J. Roentgenol..

[15] Fujiki Y (2018). Evaluation of clinical prognostic factors for interstitial pneumonia in anti-MDA5 antibody-positive dermatomyositis patients. Mod. Rheumatol..

[16] Hellmich B (2007). EULAR recommendations for conducting clinical studies and/or clinical trials in systemic vasculitis: Focus on anti-neutrophil cytoplasm antibody-associated vasculitis. Ann. Rheum. Dis..

[17] Mukhtyar C (2009). Modification and validation of the Birmingham Vasculitis Activity Score (version 3). Ann. Rheum. Dis..

[18] Guillevin L (2011). The Five-Factor Score revisited: Assessment of prognoses of systemic necrotizing vasculitides based on the French Vasculitis Study Group (FVSG) cohort. Medicine (Baltimore).

[19] Devasia RA (2009). Fluoroquinolone resistance in *Mycobacterium tuberculosis*: The effect of duration and timing of fluoroquinolone exposure. Am. J. Respir. Crit. Care Med..

[20] Kotani T (2016). Initial limited three-level thin-section computed tomography scorings predict the prognosis of acute/subacute interstitial pneumonia in patients with dermatomyositis. Mod. Rheumatol..

[21] Raghu G (2011). An official ATS/ERS/JRS/ALAT statement: Idiopathic pulmonary fibrosis: Evidence-based guidelines for diagnosis and management. Am. J. Respir. Crit. Care Med..

[22] Arancibia F (2002). Community-acquired pneumonia due to gram-negative bacteria and *Pseudomonas aeruginosa*: Incidence, risk, and prognosis. Arch. Intern. Med..

[23] Yamazaki R (2016). Clinical features and outcomes of IPF patients hospitalized for pulmonary infection: A Japanese cohort study. PLoS ONE.

[24] Yamagata M (2016). Prevalence and responsiveness to treatment of lung abnormalities on chest computed tomography in patients with microscopic polyangiitis: A multicenter, longitudinal, retrospective study of one hundred fifty consecutive hospital-based japanese patients. Arthritis Rheumatol..

[25] Ando Y, Okada F, Matsumoto S, Mori H (2004). Thoracic manifestation of myeloperoxidase-antineutrophil cytoplasmic antibody (MPO-ANCA)-related disease. CT findings in 51 patients. J. Comput. Assist. Tomogr..

[26] Gayraud M (2001). Long-term followup of polyarteritis nodosa, microscopic polyangiitis, and Churg-Strauss syndrome: Analysis of four prospective trials including 278 patients. Arthritis Rheum..

[27] Ozaki S (2012). Severity-based treatment for Japanese patients with MPO-ANCA-associated vasculitis: The JMAAV study. Mod. Rheumatol..

[28] Weiner MG (2018). POINT: Is ICD-10 diagnosis coding important in the era of big data? Yes. Chest.

