# Supplementary material for: Evaluation of poor prognostic factors of respiratory related death in microscopic polyangiitis complicated by interstitial lung disease
Source: Sci Rep. 2021 Jan 15;11:1490. doi: 10.1038/s41598-021-81311-7 (PMC7810976; doi:10.1038/s41598-021-81311-7)
Supplement: Supplementary file 1 — Supplementary Information. [file 41598_2021_81311_MOESM1_ESM.docx]

Supplementary Information for:

**Evaluation of poor prognostic factors of respiratory related death in microscopic polyangiitis complicated by interstitial lung disease**

Shogo Matsuda, ^1^ Takuya Kotani,^1*^ Takayasu Suzuka,^1^ Takao Kiboshi,^1^ Keisuke Fukui,^2^ Minako Wakama,^1^ Takaaki Ishida,^1^ Youhei Fujiki,^1^ Hideyuki Shiba,^1^ Koji Nagai,^1^ Kenichiro Hata,^1^ Takeshi Shoda,^3^ Yuri Ito,^2^ Shigeki Makino,^1^ Tohru Takeuchi,^1^

^1^Department of Internal Medicine (IV), Osaka Medical College, Takatsuki, Osaka, Japan

^2^Department of medical Statistics, Research and Development Center, Osaka Medical College, Takatsuki, Osaka

^3^Department of Rheumatology, Yodogawa Christian Hospital, Osaka, Japan

**Correspondence and reprint requests to** Takuya Kotani, MD, PhD

Department of Internal Medicine (IV), Osaka Medical College, Daigaku-Machi 2-7, Takatsuki, Osaka 569-8686, Japan

Tel.: +81-72-683-1221; Fax: +81-72-683-1801

E-mail: [in1242@osaka-med.ac.jp](mailto:in1242@osaka-med.ac.jp)

This supplement contains:

Supplementary Table S1

| **Supplementary Table S1.** Comparison of systemic symptoms and contents of treatment between MPA with or without ILD | | | | | |  |  |
| --- | --- | --- | --- | --- | --- | --- | --- |
| Characteristics | MPA without ILD (n= 33) | | | MPA with ILD (n=47) | *P* value | | |
| Systemic Symptoms | |  |  | |  | |  |
| General, n (%) | | 24 (72.7) | 31 (66) | | 0.627 | |  |
| Cutaneous, n (%) | | 8 (24.2) | 2 (4.3) | | 0.013* | |  |
| Mucous membrane, n (%) | | 5 (15.2) | 5 (10.6) | | 0.733 | |  |
| Ear, nose, throat, n (%) | | 9 (27.3) | 13 (27.7) | | 1.000 | |  |
| Chest, n (%) | | 13 (39.4) | 10 (21.3) | | 0.087 | |  |
| Alveolar hemorrhage, n (%) | | 4 (12.1) | 7 (14.9) | | 1.000 | |  |
| Cardiovascular, n (%) | | 2 (6.1) | 1 (2.1) | | 0.566 | |  |
| Abdominal, n (%) | | 1 (3.0) | 0 (0) | | 0.413 | |  |
| Renal, n (%) | | 29 (87.9) | 29 (61.7) | | 0.012* | |  |
| Nervous system, n (%) | | 16 (48.5) | 22 (46.8) | | 1.000 | |  |
| Initial treatment | |  |  | |  | |  |
| PDN (n=80), mg/day | | 50 (40-60) | 45（35-60） | | 0.158 | |  |
| MPDN pulse, n (%) | | 10（30.3） | 7 (14.9) | | 0.164 | |  |
| Immunosuppressants | |  |  | |  | |  |
| IVCY, n (%) | | 15（45.5） | 15 (31.9) | | 0.247 | |  |
| Total IVCY dose (g) | | 0.6 (0.2-1.6) | 1.5 (0.9-2.4) | | 0.013* | |  |
| RTX, n (%) | | 5 (15.2) | 4 (8.5) | | 0.477 | |  |
| IVIG, n (%) | | 3 (9.1) | 0 (0) | | 0.066 | |  |
| AZA/MTX/MMF/TAC/MZB, n (%) | | 21(63.6)/2(6.1)/1(3.0)  /1(3.0)/2(6.1) | 35(74.5)/1(2.1)/1(2.1)  /4(8.5)/2(4.3) | | 0.33/0.566/1.00/0.394/1.000 | |  |
| Apheresis | |  |  | |  | |  |
| Plasma exchange, n (%) | | 4 (12.1) | 3 (6.4) | | 0.439 | |  |

The laboratory markers are presented as the median (interquartile range). The P-values were estimated using Fisher’s exact test or Wilcoxon rank sum test. *P < 0.05. MPA: microscopic polyangiitis; ILD: interstitial lung disease; PDN: prednisolone; MPDN: methylprednisolone; IVCY: intravenous cyclophosphamide; RTX: rituximab; IVIG: intravenous immunoglobulin; AZA: azathioprine; MTX: methotrexate; MMF: mycophenolate mofetil; TAC: tacrolimus; MZB: mizoribine.

This supplement contains:

Supplementary Table S2

| **Supplementary Table S2.** Comparison of disease severity, and contents of treatment between survivors and non-survivors in MPA with ILD | | | |
| --- | --- | --- | --- |
| Characteristics | Survivors (n= 35) | Non-survivors (n=12) | *P* value |
| BVAS at onset | 12 (8-21) | 16.5 (6.5-20.8) | 0.76 |
| Five factor score 2009 |  |  |  |
| ≦1 | 10 (28.6) | 1 (8.3) | 0.244 |
| 2 | 20 (57.1) | 9 (75) | 0.324 |
| ≧3 | 5 (14.3) | 2 (16.7) | 1.000 |
| EUVAS-defined disease activity |  |  |  |
| Localized | 2(5.7) | 0(0) | 1.00 |
| Early systemic | 7(20.0) | 1(8.3) | 0.66 |
| Systemic | 19 (54.3) | 8 (66.7) | 0.517 |
| Severe | 7 (20) | 3 (25) | 0.7 |
| Initial treatment |  |  |  |
| PDN (n=47), mg/day | 45 (35-60) | 42.5 (25.6-60.5) | 0.440 |
| MPDN pulse, n (%) | 4（11.4） | 3 (25) | 0.350 |
| Immunosuppressants |  |  |  |
| IVCY, n (%) | 13（37.1） | 2 (16.7) | 0.288 |
| Total IVCY dose (g) | 1.5 (0.95-2.2) | 1.6 (0.7-2.5) | 0.799 |
| RTX, n (%) | 3 (8.6) | 1(8.3) | 1.000 |
| AZA/MTX/MMF/TAC/MZB, n (%) | 26(74.3)/1(2.9)/0(0)  /3(8.6)/2(5.7) | 9(75.0)/0(0)/1(8.3)  /1(8.3)/0(0) | 1.0/1.0/0.26/1.0/1.0 |
| Apheresis |  |  |  |
| Plasma exchange, n (%) | 2 (5.7) | 1(8.3) | 1.000 |

The laboratory markers are presented as the median (interquartile range). The P-values were estimated using Fisher’s exact test or Wilcoxon rank sum test. *P < 0.05. MPA: microscopic polyangiitis; ILD: interstitial lung disease; BVAS: Birmingham Vasculitis Activity Score; EUVAS: European Vasculitis Study Group; PDN: prednisolone; MPDN: methylprednisolone; IVCY: intravenous cyclophosphamide; RTX: rituximab; AZA: azathioprine; MTX: methotrexate; MMF: mycophenolate mofetil; TAC: tacrolimus; MZB: mizoribine.

This supplement contains:

Supplementary Table S3

**Supplementary Table S3.** Cox regression analysis of respiratory-related deaths in MPA with ILD

|  | Unadjusted |  |  |  | Adjusted | |  |
| --- | --- | --- | --- | --- | --- | --- | --- |
| Risk Factors | Hazard ratio | 95% CI | *P* |  | Hazards ratio | 95% CI | *P* |
| %FVC (for 1%) | 0.95 | 0.90-0.99 | 0.02* |  | 0.98 | 0.93-1.01 | 0.20 |
| Total GGO score | 1.26 | 1.04-1.54 | 0.02* |  | 1.21 | 0.98-1.50 | 0.08 |
| Right middle lobe FS | 2.71 | 0.84-8.95 | 0.09 |  | 1.84 | 0.44-7.16 | 0.39 |
| Right Lower lobe FS | 20.6 | 4.95-110.5 | <0.0001*** |  | 12.4 | 2.88-69.4 | 0.0005*** |
| Left Lower lobe FS | 2.82 | 1.25-6.42 | 0.01* |  | 4.65 | 1.55-17.1 | 0.0045** |
| Total FS | 1.29 | 1.04-1.60 | 0.02* |  | 1.26 | 0.98-1.63 | 0.07 |

The Hazard ratios of respiratory related death were derived from univariate and multivariate analysis with a cox regression model.

Propensity score adjustment was used for each of the risk factors in multivariate model.

Covariates: age, sex, the history of smoking, the initial dose of prednisolone, and the duration time of ILD.

*P < 0.05, **P < 0.01, ***P < 0.001. MPA: microscopic polyangiitis; ILD: interstitial lung disease; CI: confidence interval; FVC: forced vital capacity; GGO: ground-glass opacity; FS: fibrosis score.
